# Neurocognitive Decrements are Present in Intellectually Superior Schizophrenia

**DOI:** 10.3389/fpsyt.2014.00045

**Published:** 2014-05-07

**Authors:** Anja Vaskinn, Torill Ueland, Ingrid Melle, Ingrid Agartz, Ole A. Andreassen, Kjetil Sundet

**Affiliations:** ^1^Department of Psychology, University of Oslo, Oslo, Norway; ^2^NORMENT Centre of Excellence/K.G. Jebsen Centre for Psychosis Research, Oslo University Hospital, Oslo, Norway; ^3^Institute of Clinical Medicine, University of Oslo, Oslo, Norway; ^4^Department of Psychiatric Research, Diakonhjemmet Hospital, Oslo, Norway

**Keywords:** neuropsychology, psychosis, cognition, IQ, schizophrenia

## Abstract

Data suggest that individuals with schizophrenia (SZ) and superior intelligence can present without specific neurocognitive deficits. However, neurocognitive decrements, defined as worse cognition than expected, have been reported in practically all SZ cases. This study investigated if neurocognitive decrements are present in intellectually superior SZ by comparing the neuropsychological profile of SZ cases with IQ-matched healthy controls (HC) across intellectual levels. Participants with SZ and HCs were stratified into three IQ-groups; intellectually low (IQ 80–95; SZ *n* = 65 and HC *n* = 13), intellectually normal (IQ = 100–115; SZ *n* = 111 and HC *n* = 115), and intellectually superior (IQ ≥ 120; SZ *n* = 20 and HC *n* = 50). A repeated measures multivariate analysis of co-variance compared performance on eight selected neuropsychological tests across IQ-strata and diagnostic group. Differences in clinical characteristics and social functioning in SZ across IQ-strata were investigated with multivariate and univariate analyses of variance. Intellectually superior SZ participants scored within normal limits, but had neurocognitive decrements compared to superior HCs. Decrements were of the same magnitude as in the low and normal IQ-strata. Levels of functional impairments and clinical characteristics in participants with SZ did not differ significantly across IQ-strata. Results indicate that neurocognitive decrements are present in intellectually superior SZ to the same extent as in intellectually low and intellectually normal SZ, supporting the notion that SZ is a neurocognitive disorder. Similar levels of social functional deficits and clinical symptoms suggest similar disease processes in SZ across intellectual level.

## Introduction

Some individuals diagnosed with schizophrenia (SZ) perform within the normal range on cognitive measures (1–3). This has prompted research into the question of whether it is possible to have SZ without neurocognitive impairment. In the 1990s and 2000s, the question was investigated in individuals with SZ who had normal or near-normal scores on intelligence or neuropsychological tests. All of the studies found neurocognitive reductions. These included a larger percentage with learning deficits in the SZ sample compared to matched healthy control (HC) participants (4), subclinical deficits and a neuropsychological profile resembling the one seen in neurocognitively impaired SZ (5), neurocognitive deficits compared to matched non-SZ clinical control participants (6), higher verbal IQ and lower performance IQ (7), or lower processing speed and memory scores (8) compared to full scale IQ-matched HCs, and executive function and attention deficits in SZ participants with preserved IQ (3). The existence of neurocognitive reductions in individuals with SZ of average cognitive abilities supports the notion that impaired neurocognition is indeed a core feature of the disorder (9).

Later, the field has moved forward to studies of SZ samples with superior intellectual abilities, asking the same question: Is there a subgroup of individuals with SZ who are free of neurocognitive impairment? Results have been mixed. Heinrichs and colleagues (10) found that persons with SZ and verbal IQ ≥ 90th percentile did not differ from HC with equally superior verbal abilities on any other neuropsychological measure, but they did require more community support. MacCabe and co-authors (11) found that a SZ group with both premorbid and current superior intelligence did not differ from HC on any neuropsychological measure, but speculated that subtle neurocognitive deficits may still be present as the profile shape resembled those generally seen in SZ. Gray et al. (12) found the same magnitude of neurocognitive differences between persons with SZ and HC across IQ-strata, including those with WASI IQ > 120. All of these studies had methodological limitations. They allowed for the inclusion of individuals with schizoaffective disorder, two of them had quite small sample sizes [(11): *n* = 10; (12): *n* = 7], and none were catchment area-based. So far the presence of neurocognitive impairment in intellectually superior SZ has not been thoroughly examined in a large, catchment area-based, representative sample of both individuals with a diagnosis of SZ and of healthy individuals. Our research setting offers such an opportunity. In addition, we wanted to avoid the inclusion of individuals with schizoaffective disorder in the SZ sample, as they are possibly characterized by better neurocognition than SZ (13).

The overall goal of the current study is to explore whether SZ with superior intellectual abilities is characterized by neurocognitive deficits or decrements. In clinical neuropsychology, cognitive impairment or deficit is usually defined based on performance compared to a healthy reference sample (norms), often two standard deviations below the mean. In SZ research, one standard deviation is often used (14). In order to capture the neurocognitive impairment of SZ, cognitive function decrements have been proposed as an alternative to deficits compared to norms (14). Decrements are said to be present when cognitive performance is worse than would be expected, e.g., compared to premorbid estimates or general intelligence. Although many individuals with SZ have normal and some above-normal intellectual or cognitive abilities, Keefe et al. (14) found that 98% of individuals with SZ performed worse than expected in various neurocognitive domains and thus were characterized as having cognitive decrements.

In the current study, individuals with SZ from different IQ-strata are compared to HC within the same IQ-stratum. Three SZ samples – with low, normal, and superior intellectual abilities, respectively – are compared with three HC samples with low, normal, and superior intellectual abilities. Such matching on intelligence avoids confounding by a general deficit, and any subsequent SZ–HC difference will be due to deficits in specific cognitive functions. We start by asking if intellectually superior SZ individuals are neuropsychologically impaired compared to standard norms. Then we ask if the intellectually superior SZ sample is significantly impaired on specific neurocognitive functions compared to HCs with similar superior intellectual abilities. We end this set of questions by asking whether the magnitude of decrements in the intellectually superior SZ sample, should they exist, differs from the magnitude of decrements seen for the two other SZ samples when they are compared to their HC groups matched for IQ-level.

Our main research question is: Does the magnitude of neurocognitive SZ–HC differences vary across IQ-strata? Since the question has been raised as to whether SZ with intact neurocognition constitutes a less severe version of the disease (5), we ask whether our three intellectually stratified SZ groups can be distinguished on illness-related features, such as symptom load, psychopharmacological treatment, illness duration, and social functioning.

## Materials and Methods

### Participants

The study was conducted within the multi-center Thematically Organized Psychosis (TOP) Study at the University of Oslo, Norway, from 2003 to 2012. Only participants with Norwegian as their first language and/or all compulsory schooling in Norway and Wechsler Abbreviated Scale of Intelligence [WASI; (15)] Full Scale IQ ≥ 70 were included in the current study. Two-hundred and sixty-one persons aged 17–60 years with a Diagnostic and Statistical Manual for Mental Disorders (fourth ed; DSM-IV) diagnosis of SZ satisfied the inclusion criteria. Twenty-two of these individuals were excluded due to clinically significant head trauma (*n* = 20), epilepsy (*n* = 1), or migraine (*n* = 1). In order to secure a representative sample, individuals with concurrent substance abuse were not excluded. However, none were assessed while under influence of substances. The final SZ sample consisted of 239 individuals recruited from hospitals in the Oslo area. HCs from the same geographical areas were recruited through national statistical records and invited by letter to participate. They were screened with an interview to capture symptoms of severe mental illness [Primary Care Evaluation of Mental disorders; PRIME-MD; (16)] and excluded if there was any information on mental, neurological or somatic disorder. The HC sample consisted of 456 individuals. The TOP study is approved by the Regional Ethics Committee and the Norwegian Data Inspectorate, and is completed in accordance with the Helsinki Declaration. All participants received oral and written information on the study and have signed informed consent.

### Classification of sample

First, the HC sample was divided into two groups of equal size (*n* = 228) using an online randomization tool (www.randomization.com). The two HC groups did not differ in WASI IQ, age, education, or sex distribution. HC group I was used as a reference group for standardizing the neurocognition scores based on their mean and standard deviation. HC group II was used in the statistical analyses.

Second, the remaining sample (HC II *n* = 228; SZ *n* = 239) was stratified into three IQ levels based on WASI Full Scale IQ: intellectually low (IQ = 80–95), intellectually normal (IQ = 100–115), and intellectually superior (IQ ≥ 120). We chose IQ ranges leaving a gap of five IQ-points between the three IQ levels to avoid overlap between groups and increase the chances of tapping true IQ-based strata. There were 65 individuals in the low SZ group (SZ-low), 111 in the normal SZ group (SZ-normal) and 20 in the superior SZ group (SZ-superior). Corresponding numbers for HCs were 13 intellectually low (HC-low), 115 normal (HC-normal), and 50 intellectually superior individuals (HC-superior). The demographic characteristics of these six groups are shown in Table 1. A multivariate analysis of variance (MANOVA) showed significant overall effects of the demographic variables. Education differed across diagnostic group (*F*_(1, 368)_ = 14.9, *p* < 0.001, η^2^ = 0.04) and intellectual level (*F*_(2, 368)_ = 26.8, *p* < 0.001, η^2^ = 0.13), whereas age (*F* = 5.5 _(1, 368)_, *p* < 0.020, η^2^ = 0.02) and sex (*F*_(1, 368)_ = 6.1, *p* < 0.014, η^2^ = 0.02) differed across diagnostic group.

**Table 1:** **Demographic characteristics in schizophrenia and healthy participant groups and clinical characteristics in participants with schizophrenia**.

	SZ-low IQ, *n* = 65	HC-low IQ, *n* = 13	SZ-normal IQ, *n* = 111	HC-normal IQ, *n* = 115	SZ-superior IQ, *n* = 20	HC-superior IQ, *n* = 50	Statistics (*F*-values)
Age	31.8 (11.7)	34.4 (12.3)	30.0 (8.4)	36.6 (10.6)	30.9 (6.4)	31.4 (8.2)	Group: 5.5*
	18–60	18–53	17–57	18–55	22–42	19–51	
Education	10.9 (1.8)	12.1 (1.3)	12.5 (2.2)	13.6 (2.3)	14.0 (2.5)	15.0 (1.8)	Group: 14.9**
	9–16	10–14	9–18	9–18	9–18	11–18	IQ: 26.8**
Gender (males/females)	39/26	6/7	66/45	49/66	15/5	27/23	Group: 6.1*
WASI IQ	88.2 (4.3)	90.4 (4.4)	107.3 (4.5)	108.6 (4.4)	126.4 (4.1)	126.0 (4.0)	IQ: 850.9**
PANSS	16.1 (5.1)	–	14.9 (5.5)^b^	–	15.4 (5.9)^e^	–	ns
Positive symptoms scale	
PANSS	16.9 (6.3)	–	15.8 (6.5)^c^	–	16.2 (6.7)^e^	–	ns
Negative symptoms scale	
IDS-C	18.7 (12.3)^a^	–	15.1 (10.8)^d^	–	16.0 (10.1)^f^	–	ns
GAF-symptoms	38.3 (9.9)	–	41.4 (10.4)	–	43.8 (10.8)	–	ns
Age of psychosis onset	23.8 (10.1)	–	23.1 (7.5)	–	23.9 (5.8)	–	ns
	*n* = 63		*n* = 108	
Illness duration	8.3 (8.3)	–	7.0 (6.2)	–	7.0 (6.2)	–	ns
	*n* = 63		*n* = 108	
Average defined daily dose antipsychotics	1.42 (1.71)	–	1.36 (0.82)	–	1.32 (1.27)	–	ns
	*n* = 56 (86%)		*n* = 103 (93%)		*n* = 15 (75%)	
Level of care (inpatient/outpatient)	21/44 (32/68%)	–	25/86 (23/77%)	–	3/17 (15/85%)	–	ns

*GAF, Global Assessment of Functioning; PANSS, Positive and Negative Syndrome Scale; IDS-C, Inventory of Depressive Symptomatology-Clinician rated*.

*^a^*n* = 49, ^b^*n* = 109, ^c^*n* = 110, ^d^*n* = 92, ^e^*n* = 19, ^f^*n* = 15*,

*p < 0.05, **p < 0.01.

### Clinical and social function assessment

The SZ sample was assessed with the Positive and Negative Syndrome Scale [PANSS; (17)] and the Inventory of Depressive Symptomatology-Clinician rated [IDS-C; (18)]. To enable analysis of antipsychotic drug treatment across antipsychotic medications, we calculated the defined daily dose (DDD), which is the assumed average maintenance dose per day for a drug used for its main indication in adults. For example, someone receiving one antipsychotic medication with half the recommended dose has a DDD of 0.5, whereas someone who receives two antipsychotic medications, each with 80% of recommended dose, gets a DDD of 1.6. Global functioning was assessed with the Global Assessment of Functioning Scale-split version (19). In this version the GAF scale is divided into one symptom and one function score. It was used in order to improve psychometric properties. Social adjustment was assessed with the Social Functioning Scale [SFS; (20)]. SFS consists of seven subscales covering areas such as social engagement, independence, prosocial activities, and work function. SFS results are given in standardized scores (mean in SZ samples = 100, standard deviation = 15). Data are presented in Tables 1 and 4.

### Neuropsychological assessment

All participants completed a battery of standard neuropsychological tests when in a clinically stable state. *Intelligence* was assessed with the four-subtest WASI. In addition, five neuropsychological tests were chosen for the present study. *Fine motor function* was assessed with the Grooved Pegboard Test (21). *Psychomotor speed* was assessed with the Wechsler Adult Intelligence Scale [third version, WAIS-III; (22)] Digit Symbol subtest, and *attention* with WAIS-III Digit Span (combined score forward and backward). *Verbal learning* was measured using the Logical Memory Test [Wechsler Memory Scale 3rd version; WMS-III; (23)]. Subtests from the Delis–Kaplan Executive Function System [D-KEFS; Delis (24)] yielded scores for *inhibition* (Color-Word Interference test; “Stroop” condition), *phonemic* (Letter fluency/FAS) and *semantic verbal fluency* (Category fluency/Animals and Boys’ Names), and *cognitive flexibility* (Category Switching/Fruits and Furniture – number of correct responses).

### Statistical analysis

The Statistical Package for the Social Sciences (IBM SPSS Statistics for Windows, Version 20.0, IBM Corp., Armonk, NY, USA) was used. A two-way repeated measures MANCOVA of the effect of group membership (SZ or HC) and intellectual level (low, normal, superior) on neurocognition was conducted. The eight standardized neuropsychological test scores were entered as dependent variables (within-subjects factor). Group (SZ or HC) and intellectual level (low, normal, superior) were the between-subject factors. A significant interaction between group membership and intellectual level will indicate that the magnitude of SZ–HC differences varies across intellectual level. Since initial analyses found sex and age to differ across diagnostic groups and intellectual level, these two variables were entered as covariates. Although the groups differed in education, it was not included as a covariate as education is linked to our two study variables (SZ and intellectual level), i.e., inherent to the groups being considered. Follow-up univariate analyses of covariance (ANCOVAs) were conducted for each neuropsychological test and effect sizes (Cohen’s *d*) for SZ–HC differences were calculated for all neuropsychological (raw) test scores using the pooled standard deviation for the IQ-stratum in question. We also provide effect sizes for overall SZ–HC differences, across IQ-strata.

An additional two-way repeated measures MANCOVA (controlling for age and sex) was conducted to investigate effects of group and intellectual level on social function as assessed with the seven SFS subscales (within-subjects factor). It was followed by univariate analyses of variance (ANCOVA) for group and intellectual level for each of the seven SFS subscales. Differences in functioning between the SZ groups assessed with GAF-f were investigated with a univariate analysis of variance (ANOVA).

Finally, the presence of any differences in symptom load between SZ-superior and the other SZ groups was investigated with a one-way MANOVA with subsequent follow-up ANOVAs in the case of significant overall effects. Variables analyzed were symptoms as assessed with PANSS, IDS-C, and GAF-s. Differences in age of onset, illness duration, and DDD of antipsychotic medications were analyzed with three separate ANOVAs. Differences in level of care, i.e., the number of participants receiving inpatient versus outpatient treatment across IQ-strata, were examined with a chi-square test for independence.

## Results

**Figure 1:**
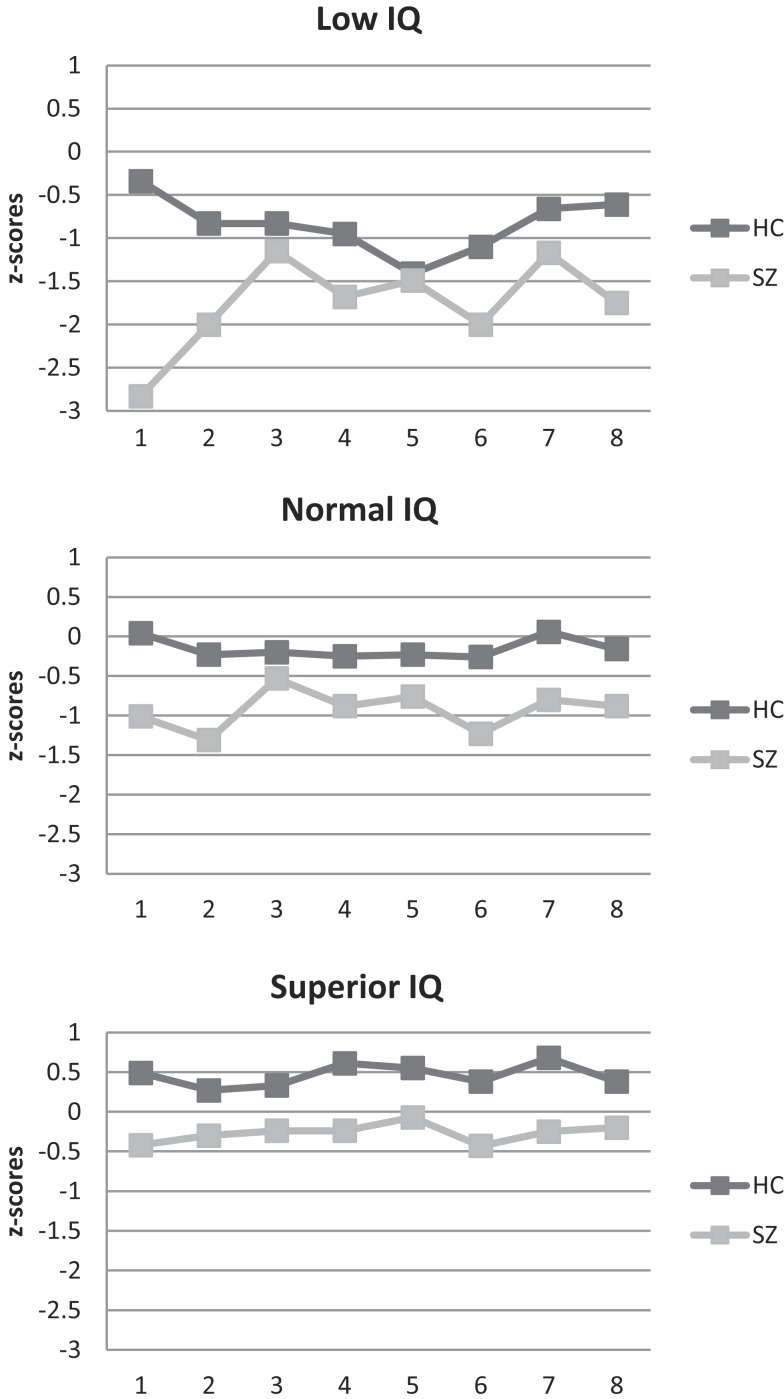
**Neuropsychological profile of schizophrenia and healthy participant groups**. (1) Fine motor function/Grooved Pegboard Test; (2) psychomotor speed/digit symbol; (3) attention/digit span; (4) Verbal learning/Logical Memory Test; (5) phonemic fluency/letter fluency; (6) semantic fluency/category fluency; (7) cognitive flexibility/category switching; (8) inhibition/“Stroop” *z*-scores were calculated based on the performance of HC group I.

The standardized neuropsychological test scores for the intellectually superior SZ sample are presented in Table 2 (and Figure 1). Although below mean, the group performs well within the normal range compared to the standardization sample (HC I).

**Table 2:** **Neuropsychological performance in participants with schizophrenia and superior intellectual abilities (*n* = 20) – standardized scores (*z*) based on the reference sample**.

	*M* (SD)
Grooved pegboard	−0.42 (0.96)
Digit symbol	−0.30 (0.89)
Digit span	−0.24 (0.77)
Logical memory learning	−0.24 (1.25)^a^
Phonemic fluency	−0.07 (0.96)
Semantic fluency	−0.43 (1.04)
Category switching	−0.25 (0.85)
Inhibition	−0.20 (1.09)

*^a^*n* = 18*.

The magnitude of neurocognitive SZ–HC differences did not differ across IQ-strata (see Table 3). The repeated measures MANCOVA for the eight standardized neuropsychological test scores yielded a significant overall main effect of neuropsychological test [*F*_(7, 350)_ = 8.0, *p* < 0.001, Wilk’s Lambda = 0.86, η^2^ = 0.14], indicating that performance differs across tests. The interaction effects between neuropsychological test and group [*F*_(7, 350)_ = 12.0, *p* < 0.001, Wilk’s Lambda = 0.81, η^2^ = 0.19] and between neuropsychological test and intellectual level [*F*_(14, 702)_ = 7.8, *p* < 0.001, Wilk’s Lambda = 0.75, η^2^ = 0.13], respectively, were significant. In other words, the neuropsychological profile differs between SZ and HC and between individuals of different intellectual levels. Finally, the interaction effect between neuropsychological test, group, and intellectual level was non-significant [*F*_(14, 700)_ = 1.1, *p* = 0.350, Wilk’s Lambda = 0.96, η^2^ = 0.02]. Differences in neurocognitive performance were consistent across IQ-strata as was the shape of the neuropsychological profile (see Figure 1). As expected, follow-up ANCOVAs yielded non-significant interaction effects (reported in Table 3). Effect sizes (see Table 3) for overall SZ–HC differences were largest for processing speed and semantic fluency, and smallest for inhibition, attention, and phonemic fluency. IQ-stratified effect sizes ranged from medium-sized to large for intellectually superior participants, from small to large for participants with normal intelligence and from small to large for intellectually low participants, using Cohen’s rules of thumb (25).

**Table 3:** **Neuropsychological performance (raw scores) in schizophrenia and healthy participant groups**.

	Low IQ IQ 80–95			Normal IQ IQ 100–115			Superior IQ IQ ≥ 120			Overall effect size SZ-HC differences across IQ-strata	ANCOVA		
	SZ-low, *n* = 65, *M* (SD) range	HC-low, *n* = 13, *M* (SD) range	Effect size SZ-HC differences	SZ-normal, *n* = 111, *M* (SD) range	HC-normal, *n* = 115, *M* (SD) range	Effect size SZ–HC differences	SZ-superior, *n* = 20, *M* (SD) range	HC-superior, *n* = 50, *M* (SD) range	Effect size SZ-HC differences		Group, *F*-value	IQ, *F*-value	Group × IQ, *F*-value
Grooved Pegboard (composite)	90.5 (35.2)^a^	69.2 (8.6)	0.97	74.9 (12.3)	65.9 (8.6)^d^	0.86	69.8 (8.2)	62.0 (6.8)	1.04	0.91	35.3**	8.4**	2.6
	60–289	59–84		57–122	48–112		58–98	50–83	
Digit symbol	51.6 (13.4)^b^	66.7 (11.5)	1.21	60.3 (13.2)	74.7 (13.2)	1.09	73.9 (12.0)	81.5 (12.7)	0.62	1.22	52.1**	29.2**	3.4
	22–82	45–83		33–101	47–111		50–93	54–109	
Digit span (composite)	9.1 (1.6)	9.8 (2.4)	0.35	10.4 (1.6)	11.0 (1.9)	0.34	11.0 (1.5)	12.1 (2.1)	0.61	0.64	10.7**	16.5**	0.5
	6–12	6–14		7–15	7–16		8–13	8–17	
Logical memory learning	17.8 (6.3)^b^	21.8 (6.3)	0.64	22.2 (7.5)^c^	25.6 (6.0)	0.50	25.7 (6.8)^e^	30.3 (6.2)	0.71	0.80	13.9**	20.9**	0.2
	6–38	6–30		8–37	12–41		15–38	14–45	
Phonemic fluency	30.1 (9.5)	30.9 (6.0)	0.10	37.8 (11.7)	43.4 (10.5)	0.50	45.1 (10.2)	51.6 (10.1)	0.64	0.76	5.5*	38.5**	1.0
	8–47	23–40		16–80	17–74		30–69	28–72	
Semantic fluency	34.2 (8.1)	40.7 (8.4)	0.79	39.7 (9.1)	47.2 (8.4)	0.86	45.9 (8.0)	52.1 (7.6)	0.80	1.06	28.3**	24.5**	0.2
	19–53	24–55		23–75	23–76		31–62	35–72	
Category switching	11.4 (2.5)	12.5 (2.5)	0.44	12.2 (2.5)	14.7 (2.8)	0.94	13.8 (2.2)	16.3 (2.3)	1.11	1.07	25.9**	20.7**	0.7
	6–17	7–17		5–18	4–23		10–17	11–23	
Inhibition	67.2 (18.6)	55.5 (8.0)	0.89	58.2 (13.7)	50.8 (10.1)	0.62	51.3 (11.2)	45.3 (8.2)	0.62	0.48	18.1**	15.0**	0.8
	35–120	38–69		34–104	31–95		35–77	30–70	

*^a^*n * = 62, ^b^*n * = 64, ^c^*n* = 108, ^d^*n* = 114, ^e^*n * = 18*.

**p < 0.01, *p < 0.05.

IQ-level had no effect on social function (see Table 4). The overall repeated measures MANCOVA for social function as assessed by the SFS yielded a significant main effect of the social function scales [*F*_(6, 341)_ = 16.5, *p* < 0.001, Wilk’s Lambda = 0.78, η^2^ = 0.23] as well as of the interaction effect between social function scales and group [*F*_(6, 341)_ = 8.1, *p* < 0.001, Wilk’s Lambda = 0.88, η^2^ = 0.13]. The interaction between social function scales and intellectual level was non-significant [*F*_(12, 682)_ = 0.9, *p* = 0.523, Wilk’s Lambda = 0.97, η^2^ = 0.02]. The interaction effect between social function scales, group, and intellectual level just reached significance with a small effect size [*F*_(12, 686)_ = 1.8, *p* = 0.041, Wilk’s Lambda = 0.94, η^2^ = 0.03]. Follow-up ANCOVAs for each of the seven SFS scales yielded only significant effects of group (SZ or HC), not of intellectual level or of the interaction between group and intellectual level (see Table 4). The results from the ANOVA for the GAF-f scores in the three SZ groups were non-significant.

**Table 4:** **Social functioning in schizophrenia and healthy participant groups**.

	SZ-low, *n* = 60	HC-low, *n* = 13	SZ-normal, *n* = 103	HC-normal, *n* = 114	SZ-superior, *n* = 18	HC-superior, *n* = 49	ANCOVA		
							Group, *F*-value	IQ, *F*-value	Group × IQ, *F*-value
SFS 1: withdrawal/social engagement	99.4 (12.4)	120.1 (7.5)	101.8 (10.3)	121.3 (8.5)	98.1 (8.7)	120.6 (9.7)	197.4**	0.9	0.7
SFS 2: interpersonal communication	109.2 (16.9)	136.9 (10.6)	114.0 (17.8)	139.6 (11.2)	118.4 (21.3)	137.3 (13.1)	110.6**	1.6	1.2
SFS 3: independence-performance	102.4 (11.8)	116.0 (9.7)	104.0 (11.8)	117.1 (8.6)	105.1 (11.3)^e^	118.6 (7.1)	68.1**	1.9	0.2
SFS 4: independence-competence	106.0 (12.8)	123.0 (0)	110.3 (11.1)	122.3 (3.2)	111.4 (10.9)	122.2 (3.1)	109.5**	1.1	1.9
SFS 5: recreation	103.7 (15.3)	127.2 (11.6)	105.2 (14.7)	126.9 (12.5)	108.8 (14.4)	124.8 (12.9)	93.6**	0.5	0.8
SFS 6: prosocial activities	101.4 (15.0)	125.3 (9.7)	106.8 (12.6)	123.8 (10.0)	102.7 (11.5)	125.3 (8.6)	148.8**	0.6	2.3
SFS 7: employment/occupation	101.7 (11.8)	121.5 (2.4)	103.6 (11.2)^b^	122.0 (2.4)^d^	107.4 (10.6)	121.1 (5.2)	211.6**	1.0	2.4
GAF-function	40.7 (8.9)^a^	–	42.9 (9.9)^c^	–	42.6 (8.9)^f^	–	–	1.1 (ANOVA)	–

*SFS, Social Functioning Scale*.

*^a^*n* = 65, ^b^*n* = 102, ^c^*n* = 111, ^d^*n* = 113, ^e^*n* = 17, ^f^*n* = 20*.

**p < 0.01, *p < 0.05.

Finally, there were no clinical differences across IQ-strata among individuals with SZ. The three SZ groups had very similar symptom scores (see Table 1), and the overall MANOVA yielded no significant group differences [*F*_(8, 294)_ = 0.7, *p* = 0.655, Wilk’s Lambda = 0.96, η^2^ = 0.02]. Similarly, no significant group differences appeared for age of onset, illness duration, daily dosage of antipsychotic medication, or in level of care, although relatively fewer persons in the superior sample used antipsychotic medications or received inpatient care.

## Discussion

This study found neurocognitive decrements in a group of intellectually superior SZ participants compared to an intellectually superior HC group. The magnitude of the decrements was similar to those found for intellectually normal and intellectually low SZ. So, answering our first research question, the magnitude of neurocognitive SZ–HC differences does not vary across IQ-strata. Our results are in line with previous findings of no variation in the overall magnitude of neurocognitive differences between SZ participants and HC of different IQ-strata (12), of similar neuropsychological profile in participants with SZ across intellectual level (11), and of reduced social functioning in SZ participants with superior intelligence (10). Overall, the current literature supports the presence of neurocognitive decrements in intellectually superior SZ, although diverging results exist (10). One reason that some previous studies (11) did not find statistically significant neuropsychological differences between SZ participants and IQ-matched HC could be small sample sizes and lack of statistical power.

None of the neuropsychological test results of the intellectually superior SZ group fell below the normal range using standardized scores ( >1 standard deviation). Thus, according to an approach typically used in clinical neuropsychology, they have no neurocognitive deficits. Therefore, one could say that it is possible to have SZ and be neuropsychologically *normal*. However, this approach masks the decrements readily visible in the figure and that our statistical approach exposed. Visual inspection of the scores in Figure 1 reveals two important findings. First, although within what is considered the normal range when compared to a normal control group not stratified based on intelligence, this intellectually superior SZ group, nevertheless, performs below the mean on all tests. Further, their performance is below what would be expected based on their IQ more than a standard deviation above the mean. This discrepancy constitutes further evidence for the existence of neurocognitive decrements in intellectually superior SZ. Consequently, it seems more correct to say that it is possible to have SZ and be neuropsychologically normal, but only if you have superior intellectual abilities – and it is not possible to be neuropsychologically superior, even if you have superior intellectual abilities.

The overall SZ–HC effect sizes are much as we know from the broader literature (26) with larger impairments for *processing speed* (Digit Symbol) and *semantic fluency* (Category fluency), and smaller impairments for *attention* (Digit Span) and *phonemic fluency* (Letter fluency/FAS). For the intellectually superior SZ sample, the largest effect sizes were seen for *cognitive flexibility* (Category Switching) and *fine motor function* (Grooved Pegboard Test). A large effect size was also seen for *semantic fluency*. This was also the test that deviated most from the mean of HC group I using standardized scores (−0.43). After *semantic fluency*, *fine motor function* showed the largest deviation from the HC I mean (−0.42), corroborating the finding from the statistical analyses. Neuromotor abnormalities (27) as well as impaired psychomotor speed are present and central in SZ (28, 29). The Grooved Pegboard test is dependent upon both skills. Our measures of *cognitive flexibility* and *semantic fluency* are subtests from the same verbal fluency measure and can be considered measures of semantic memory that depend upon accessing the semantic store. Findings of impaired category fluency are robust in SZ (30). This impairment could be due to problems with the store (semantic memory) or with accessing it (retrieval which involves executive functioning). Persons with SZ show abnormal clustering on category fluency tasks suggesting a degraded store. On the other hand, similar levels of impairment in phonemic and semantic fluency indicate executive impairment, as does slow or reduced switching between categories – both of which are seen in SZ samples in general (30) as well in our sample (medium-to-large effect size for *phonemic fluency*). Thus, impaired semantic memory in SZ involves executive function. In other words, the superior sample in our study has, in spite of their superior IQ, neurocognitive problems that are very common in individuals with SZ – with motor function, speeded processing, semantic memory, and executive function. A recent large review and meta-analysis of cognitive studies from around the world, spanning several decades (26), found the largest impairments for episodic memory, speed of processing and semantic fluency, and relatively smaller impairments for measures of intelligence. We found this pattern to hold true within IQ-stratified groups as well as for the overall sample, across IQ-strata.

Our second research question concerned differences in illness-related features across IQ-strata within the SZ sample. We found no evidence of such differences. Similar functional impairments, similar levels of both psychotic and depressive symptoms and of daily dosage of antipsychotic medications were observed. Thus, our results are in line with previous reports indicating weak associations between neurocognition and clinical symptoms (31). It has been proposed that the SZ disease process could differ between individuals who differ in neurocognitive performance (32) and that neurocognitively intact SZ constitutes a less severe form of the disease (5). We found no support for such hypotheses, although we do acknowledge that there were indeed fewer in the intellectually superior SZ sample that used antipsychotic medications or who received treatment in inpatient facilities. In spite of this, having intellectual abilities well above the population mean does not offer protection against the everyday problems and the suffering associated with SZ. Thus, although they have superior intellectual abilities, this group seems unable to utilize this asset by transferring it to better coping with the challenges of everyday life. The results of an early study on monozygotic twins discordant and concordant for SZ are in line with this finding (33). In that study, levels of social and vocational impairments were similar and reduced in all affected cases, but intact in non-affected discordant monozygotic twins. However, subtle neurocognitive impairments were found in the non-affected twins. One interpretation of such findings is that social impairments are a disease-specific factor, whereas impairments in cognition might be a genetic vulnerability marker. In our cognitively diverse SZ sample, there must be other predictors of functioning besides neurocognition at play. Indeed, neurocognition does not always predict functional outcome, as we have previously shown for a non-overlapping first-episode psychosis sample (34). Other possible predictors, known to impact functioning, are of an internal nature such as social cognition (35) or dysfunctional beliefs (36). External, societal factors such as availability of health and employment services may also influence functioning. These factors were not investigated in the current study.

Limitations of this study include the cross-sectional design and the lack of information on premorbid intellectual function. Intellectual decline has been demonstrated for SZ (37), and it is quite possible that our participants have deteriorated from premorbid levels. Further, as the study addressed group comparisons, the existence of a single individual with comparable and superior neurocognitive and intellectual abilities is possible. Additionally, we only included treatment-seeking individuals with SZ. The existence of a separate subgroup of very high-functioning individuals with SZ that are not in contact with the mental health services, and that we therefore were not able to get hold of given our recruitment strategy, cannot be ruled out. Also, our subtyping approach yielded unequal cell sizes with few cases in the low (HC) and superior (SZ) IQ-strata. This is a limitation of the study, but the rule-of-thumb of having more cases in each cell than number of dependent variables was not violated (13 participants in the cell with the fewest cases versus eight neuropsychological tests). Another statistical concern is that our results are simply a reflection of regression toward the mean. Although we cannot be sure that this is not the case, we also note that the idea of regression to the mean as a causal explanation for change or difference in scores has been heavily criticized from a statistical standpoint and even called out as a myth (38). According to these authors, the idea that a second score will invariably be less extreme than the first score (in our case that a person with SZ who was selected on the basis of an extremely high IQ score will automatically present with lower neuropsychological test scores) has taken the form of a law. They warn of treating regression toward the mean as a causal agent on an individual’s score. Strengths of our study include the use of a catchment area-based approach for the recruitment of a large representative sample of both healthy and ill individuals, the exclusion of individuals with schizoaffective disorder, the matching of HC based on IQ-strata, and assessment with a large neuropsychological test battery.

In summary, our study supports the view that people with SZ and superior intelligence have neurocognitive decrements when compared to a relevant control group, i.e., matched on IQ. Therefore, the study also supports the notion of the “primacy of cognition in SZ” (39). Further, similar levels of symptoms and functional impairments across the intellectual spectrum indicate that intellectually superior SZ is not a separate disease entity or a less severe type of SZ. Such findings lend support to the idea that cognition is a central characteristic of the disorder and that using neurocognition as a biomarker in genetic studies is warranted. The findings also underline the importance of looking at relative decrements, even in persons with superior abilities. The door is open for exploration of whether cognitive-enhancing treatment could be useful also for this group.

## Conflict of Interest Statement

The authors declare that the research was conducted in the absence of any commercial or financial relationships that could be construed as a potential conflict of interest.
